# First report of the invasive mosquito *Aedes koreicus* (Diptera: Culicidae) and of its establishment in Liguria, northwest Italy

**DOI:** 10.1186/s13071-019-3589-2

**Published:** 2019-07-05

**Authors:** Marco Ballardini, Stefano Ferretti, Giorgio Chiaranz, Alessandra Pautasso, Maria Vittoria Riina, Giorgia Triglia, Federica Verna, Veronica Bellavia, Maria Cristina Radaelli, Enrica Berio, Annalisa Accorsi, Marina De Camilli, Umberto Cardellino, Nicolò Fiorino, Pier Luigi Acutis, Cristina Casalone, Walter Mignone

**Affiliations:** 1 Istituto Zooprofilattico Sperimentale del Piemonte, Liguria e Valle dʼAosta (IZSPLV), Turin, Italy; 2 Centro Studi BioNaturalistici S.r.l, Genoa, Italy; 3 Il Rastrello Cooperativa Sociale a r. l. - Onlus, Genoa, Italy; 4 Istituto Zooprofilattico Sperimentale del Piemonte, Liguria e Valle d’Aosta (IZSPLV), Imperia, Italy; 5 Ufficio Animali, Comune di Genova, Genoa, Italy; 6 ASL 3 Genovese, Genoa, Italy

**Keywords:** Invasive mosquitoes, *Aedes*, Entomological surveillance, Early detection

## Abstract

**Background:**

Invasive mosquito species (IMS) of the genus *Aedes* are a cause of increasing concern in Europe owing to their ability to vector important human viral diseases. Entomological surveillance to early detect alien mosquito and flavivirus circulation in Liguria, northwest Italy, has been carried out since 2011.

**Results:**

The invasive species *Aedes koreicus* was first detected in Genoa in September 2015, when a male specimen was caught near the international airport; species identity was confirmed by genetic analysis. Over the next three years, 86 more adult specimens were trapped at sites throughout the city, accounting for 0.50% of all mosquitoes and 1.04% of *Aedes* sp. mosquitoes trapped in Genova in the four-year period 2015–2018. So far, no other monitored sites in Liguria have revealed the presence of this species. Ovitraps at two sites became positive for the species in July–August 2017. All female *Ae. koreicus* pools analysed were negative in biomolecular assays for *Flavivirus*.

**Conclusions:**

Our findings of *Ae. koreicus* in Genoa constitute, to the best of our knowledge, the first report of the species in northwest Italy and in a Mediterranean port city. The species appears to be established; trapping and climatic data support survival of *Ae. koreicus* in the area through three consecutive winters. Monitoring of adult mosquitoes detected the species two years before its discovery with ovitraps; trapping for adult specimens appears to be a more effective tool for the early detection of IMS. The airport (located near the commercial port area) and the flower market are the most probable sites of introduction; however, the exact time and place of arrival of this IMS in Liguria remain unknown. Based on morphological and genetic data, a common origin for most of the *Ae. koreicus* populations established in Europe is suspected. So far, no control measures have been adopted in Genoa and the species will probably colonize an even wider area in the next few years.

## Background

Invasive mosquito species (IMS), particularly of the genus *Aedes*, are a source of increasing concern in Europe because of their ability to spread into new territories and cause outbreaks of human viral diseases, such as dengue, Zika, chikungunya, yellow fever and Japanese encephalitis [1]. The Asian tiger mosquito *Aedes albopictus*, endemic to southeast Asia, was the first invasive exotic mosquito species to become established in Europe, where it was first reported in Albania in 1979 [2] and in Italy in 1990 [3]. It was responsible for the first European outbreak of chikungunya fever in Emilia-Romagna, Italy, in 2007 [4], and for the 2017 outbreak in central and southern Italy [5]. Several more alien mosquito species can be found in Europe, including *Ae. aegypti* (established in Madeira and on the Eastern Black Sea coast), *Ae. atropalpus*, *Ae. japonicus* and *Ae. koreicus* [6], the latter two being recently found in Italy [7, 8].

Originally endemic to southeast Asia, *Ae. koreicus* was first found in Belgium in 2008, which was the first report of this species outside its native range; it established in a small area of 6 km^2^ and does not seem to be colonizing the surrounding territory [9]. In 2011, it was found in northeast Italy [7], where it has rapidly expanded its range [10–12], and later in Germany [13], along the Swiss-Italian border [14], in Hungary [15] and Slovenia [16]. Most of the *Ae. koreicu*s specimens found in Europe are morphologically consistent with the species phenotype characteristic of Jeju-do Island, located south of Korea, as they carry a basal band of clear scales on hind tarsomere V, which is absent in mainland specimens [7, 9]. The sole exception is a single population found in Germany in 2016–2017, whose morphological features differ, which seems to have originated from an independent introduction, possibly from mainland Korea [17].

Importation routes in Europe for this species remain unknown [18]; however, as most likely happened with other invasive *Aedes* spp., it might have been introduced at the egg or larval stage through the trade of used tyres or plants [19]. *Aedes koreicus* is able to produce cold- and drought-resistant eggs, allowing it to survive winter temperatures; moreover, as it tolerates lower temperatures better than the congeneric *Ae. albopictus*, it is active earlier during the season and can occupy an empty niche at higher altitudes [10]. Given its strong morphological similarity with *Ae. japonicus japonicus*, *Ae. koreicus* might go undetected in areas already invaded by the latter, as happened in Slovenia [16]. Genetic confirmation is needed when specimens are damaged, or mosquito collection is performed in new areas. Several studies have highlighted the potential role of *Ae. koreicus* as a vector of viral and parasitic diseases. Field-collected *Ae. koreicus* specimens were found infected by the Japanese encephalitis virus [20] and the dog heartworm *Dirofilaria repens* [21]. Moreover, the species was experimentally proven to be a competent vector of chikungunya virus [12] and of *D. immitis* [22, 23]. Entomological and virological surveillance programs are therefore extremely helpful in the early detection of IMS and of the circulation of mosquito-borne viruses and for the rapid deployment of control measures by local health authorities.

Starting in 2011, our institute (IZSPLV) has carried out entomological and virological surveillance of mosquitoes in northwest Italy (Piedmont, Liguria and Valle dʼAosta) within the framework of research projects funded by the Italian Ministry of Health and the European Union. In 2016, due to the global Zika emergency, mosquito surveillance in Liguria was intensified through the support of the regional government and the collaboration of the public Health Services (AASSLL) and the City of Genova.

This study reports the finding of *Ae. koreicus* in Genoa over four consecutive years. It is, to the best of our knowledge, the first report of the species in northwest Italy and in a port city of the Mediterranean basin. It provides clear evidence of the establishment of *Ae. koreicus* in the area, which may herald further spread of this IMS in Italy and beyond.

## Methods

### Adult mosquito collection

**Fig. 1:**
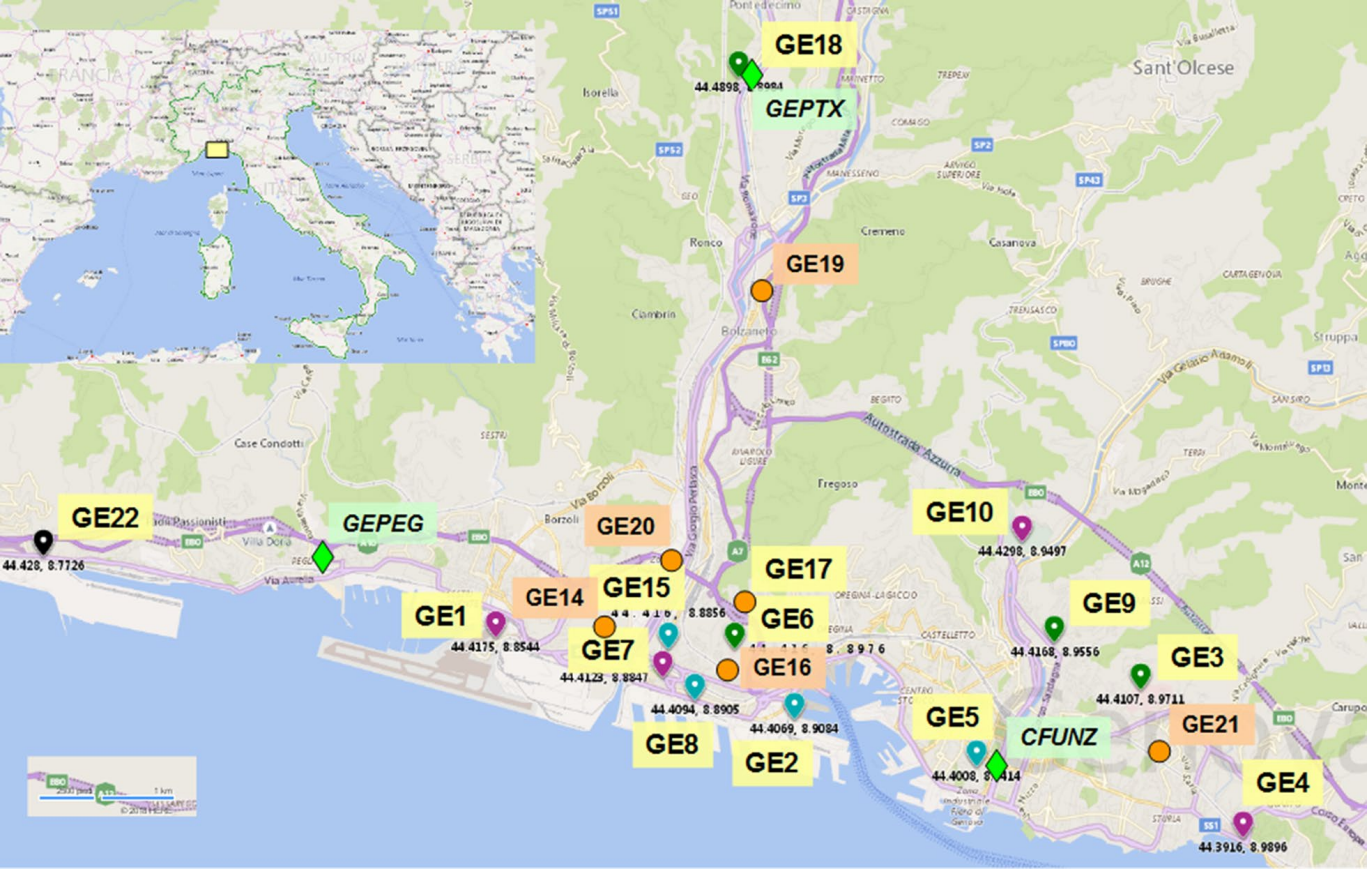
Traps with/without associated ovitraps (yellow), ovitraps only (orange) and weather stations (green) in Genoa (map published under the Microsoft® Bing™ Maps Platform APIs’ Terms of Use)

In the four-year period 2015–2018, adult mosquitoes were trapped fortnightly by trained operators, by means of CO_2_-lure-baited BG-sentinel traps (Biogents, Regensburg, Germany) and hay infusion-baited gravid traps (John Hock company, Gainesville, FL, USA) in sites throughout Liguria (*n* = 11 in 2015, *n* = 23 in 2016 and 2017, *n* = 21 in 2018). Trapping sites were chosen according to risk factors, i.e. the possible introduction of alien mosquito species (ports, airports, commercial hubs) or the potential circulation of endemic and/or imported mosquito-borne viruses, such as West Nile virus [24] or dengue, Zika and chikungunya viruses [25] (hospitals with infectious diseases departments, city neighbourhoods with a high concentration of residents from South America, where several mosquito-borne human diseases are endemic). Surveillance was increased from 2016 onwards, due to the concurrent worldwide Zika epidemics. Particular attention was directed to Genoa, where the number of monitored sites was increased from four in 2015 to 10–12 in 2016–2018 (Fig. 1).

Genoa is an important commercial and tourist hub in the Mediterranean basin and has a considerable resident community of immigrants from South America. The duration of the monitoring season and the number of night traps at each site in Genoa varied depending on the year.

At each session, the traps worked for approximately 24 consecutive hours; caught mosquitoes were or cooled to 0–4 °C or transported alive to the closest IZSPLV branch, where they were kept frozen and sent to the IZSPLV entomological lab in Imperia for processing. All mosquitoes were identified to species level according to morphological keys [26–28].

Doubtful *Aedes* spp. specimens were genetically identified by sequencing a fragment of the mtDNA *nad*4 gene using two primer sets, one for the analysis of all species within the genus *Aedes* (N4J-8502D/N4N-8944D) [29] and one specific for the identification of *Ae. koreicus* (ND4KorF/N4N-8944D) [30]. Expected fragment sizes corresponded to 440 and 283 bp, respectively. The PCR protocol was the same for both reactions and was carried out in a 25 μl final volume containing Platinum™ quantitative PCR SuperMix-UDG 2x (Thermo Fisher, Waltham, MA, USA), 0.3 uM of each primer (Metabion, Steinkirchen, Germany) and 10–50 ng of genomic DNA.

Both forward and reverse reads were sequenced on an ABI Prism 3100 Genetic Analyzer (Applied Biosystems, Waltham, MA, USA), and assembled with DNASTAR Seqman software. The consensus sequences were then compared with those deposited in GenBank (NCBI) and species assignment was based on a minimum similarity value of 98%. Consensus obtained with the *Ae. koreicus* specific primer set was also screened for the characteristic single nucleotide polymorphism (SNP), where a T is present in *Ae. koreicus*, a G in *Ae. japonicus*, and the other *Aedes* spp. have an A [30].

Female specimens underwent a biomolecular essay to detect flaviviral infection: an RT-PCR targeting the NS5 gene locus of the *Flavivirus* genome [31].

After the initial detection of *Ae. koreicus* in 2016, a retrospective investigation of samples collected in 2015 was also performed based on: (i) visual examination of a photographic database of damaged *Aedes* spp. specimens; and (ii) a biomolecular assay for the mtDNA *nad*4 locus on damaged *Aedes* spp. archival specimens.

### *Aedes* spp. egg collection

Between 2015 and 2018, standard *Aedes* mosquito ovitraps (12 cm upper diameter black vases filled with 1000 ml of water and equipped with masonite™ strips as oviposition support) were deployed at various sites throughout Liguria and monitored biweekly. The number and location of the ovitraps located in Genoa varied depending on the sampling year (*n* = 4 in 2015, *n* = 13 in 2016, *n* = 15 in 2017, *n* = 12 in 2018) and included five sites not previously surveyed for adult mosquitoes (Fig. 1).

After each monitoring session, the masonite™ strips and water in the ovitraps were collected and transported to the closest IZSPLV branch, where they were kept at room temperature and sent to the IZSPLV entomological lab in Imperia for mosquito rearing. Water was collected in order not to miss any mosquito larvae that could have already hatched. *Aedes* eggs and larvae were counted and reared at room temperature and fed with ground dog/cat food to obtain fourth-instar larvae/adult specimens for species identification based on morphological characters.

### Climatic data

Climatic data, comprising monthly average and absolute minimum air temperature and precipitation (cumulated rain) data recorded at three sites representative of three different areas of Genoa (west, inland, city centre), were analysed and compared with mosquito trapping data from the four-year period July 2015 to October 2018. The sites were located in: Genoa Pegli (GEPEG; 44.4323N, 8.8246E, 69 meters above sea level, masl), Genoa Pontedecimo (GEPTX; 44.4885N, 8.9001E, 75 masl), and Genoa Centro Funzionale (CFUNZ; 44.4004N, 8.9459E, 30 masl), respectively.

Climatic data were obtained from the weather and climatic online database run by the Osservatorio Meteo Idrologico della Regione Liguria (OMIRL), based on data collected by the Agency for the Protection of the Environment of Liguria (ARPAL) (http://www.cartografiarl.regione.liguria.it/SiraQualMeteo/script/PubAccessoDatiMeteo.asp).

## Results

### Adult mosquitoes

*Ae. koreicus* monthly capture data and number of trap nights for each site monitored in Genoa are shown in Tables 1 and 2, respectively. A total of 4499 adult mosquitoes, including 1862 *Aedes* spp., were trapped in Genoa between June and October 2016. On 29th and 30th June, two female *Aedes* sp. specimens morphologically analogous to *Ae. japonicus*/*Ae. koreicus* in several diagnostic features were trapped at two sites in the Sampierdarena quarter by means of a BG-sentinel trap (site GE7) and a gravid trap (site GE6), respectively. The two sites are located 1.1 km apart. The presence of a clear band at the base of the fourth tarsomere of the hind legs confirmed their species identification as *Ae. koreicus.* A further clear band on tarsomere V of the hind legs was consistent with the same phenotype already detected in most of the other European sites, including northeast Italy. Further morphological evidence of their species identification was obtained by visual comparison of the two Ligurian specimens with some *Ae. koreicus* collected in Trentino, northeast Italy.

**Table 1:** *Aedes koreicus* monthly capture data for each site monitored in Genoa (July 2015 to October 2018)

Site ID	Site	Coordinates	2015 (July–October)			2016 (June–October)			2017 (May–October)			2018 (June–October)		
			Trap type	Trap nights	*N*/trap night^a^	Trap type	Trap nights	*N*/trap night	Trap type	Trap nights	*N*/trap night	Trap type	Trap nights	*N*/trap night
GE1	Airport	44.4175N, 8.8544E	GT	7	0.14	BGT	9	0.00	GT	9	0.00	BGT	9	0.00
GE2	Port	44.4069N, 8.9084E	BGT	7	0.00	BGT	6	0.00	BGT	3	0.33	BGT	10	0.00
GE3	Hospital	44.4107N, 8.9711E	GT	8	0.00	GT	9	0.00	GT	11	0.55	GT	9	0.22
GE4	Hospital	44.3916N, 8.9896E	GT	9	0.00	GT	9	0.00	GT	7	0.00	GT	9	0.00
GE5	Hospital	44.4008N, 8.9414E	–	–	–	GT	10	0.00	GT	10	0.10	GT	9	0.11
GE6	Hospital	44.4160N, 8.8976E	–	–	–	GT	8	0.88	GT	12	1.25	GT	8	4.25
GE7	City centre	44.4123N, 8.8847E	–	–	–	BGT	8	0.38	BGT	7	0.14	BGT	10	0.00
GE8	City centre	44.4094N, 8.8905E	–	–	–	BGT	9	0.00	BGT	1	0.00	–	–	–
GE9	City centre	44.4168N, 8.9556E	–	–	–	BGT	8	0.00	–	–	–	–	–	–
GE10	Cemetery	44.4298N, 8.9497E	–	–	–	BGT	10	0.00	BGT	4	0.00	BGT	10	0.00
GE15	City centre	44.4160N, 8.8856E	–	–	–	–	–	–	GT	8	0.25	–	–	–
GE17	Cemetery	44.4210N, 8.9023E	–	–	–	BGT	1	0.00	BGT	4	0.25	–	–	–
GE18	Flower Market	44.4898N, 8.8984E	–	–	–	–	–	–	GT	10	0.80	GT	6	0.67
GE22	Port	44.4280N, 8.7726E	–	–	–	–	–	–	–	–	–	BGT	10	0.00

^a^N, Number of *Aedes koreicus*

*Abbreviations*: GT, gravid trap; BGT, BG-sentinel trap

**Table 2:** Trapping (number of trap nights) in Genoa in the four-year period 2015–2018

Year	Month	GE1	GE2	GE3	GE4	GE5	GE6	GE7	GE8	GE9	GE10	GE15	GE17	GE18	GE22
2015	7	2	2	2	2	–	–	–	–	–	–	–	–	–	–
2015	8	1	2	2	2	–	–	–	–	–	–	–	–	–	–
2015	9	3	2	2	2	–	–	–	–	–	–	–	–	–	–
2015	10	2	1	2	3	–	–	–	–	–	–	–	–	–	–
2016	6	2	2	2	2	2	2	2	2	1	2	–	–	–	–
2016	7	1	2	2	2	2	2	2	2	2	2	–	–	–	–
2016	8	2	1	1	2	2	2	2	2	2	2	–	1	–	–
2016	9	2	1	2	2	2	2	1	2	2	2	–	–	–	–
2016	10	2	0	2	1	2	0	1	1	1	2	–	–	–	–
2017	5	2	0	1	1	2	2	1	0	–	0	2	–	1	–
2017	6	1	0	2	2	2	2	2	0	–	0	2	–	2	–
2017	7	0	0	2	1	2	2	1	0	–	0	2	–	2	–
2017	8	2	0	2	1	1	2	0	0	–	0	2	–	1	–
2017	9	2	2	2	0	2	2	1	0	–	2	0	–	2	–
2017	10	2	1	2	2	1	2	2	1	–	2	0	–	2	–
2018	6	2	2	2	2	2	2	2	–	–	2	–	–	2	2
2018	7	2	2	2	2	2	1	2	–	–	2	–	–	2	2
2018	8	2	2	1	1	1	1	2	–	–	2	–	–	1	2
2018	9	2	2	3	3	3	3	2	–	–	2	–	–	1	2
2018	10	1	2	1	1	1	1	2	–	–	2	–	–	0	2

*Abbreviations*: GE1, airport; GE2; port; GE3, hospital; GE4, hospital; GE5, hospital; GE6, hospital; GE7, city center; GE8, city centre, GE9, city centre; GE10, cemetery; GE15, city centre; GE17, cemetery; GE18, flower market; GE22, port

For both samples from Liguria, analysis with the N4J8502D/N4N-8944D primer set showed an identity of 99% with *Ae. koreicus* sequences deposited in GenBank (KT945239.1, JF430392.1). Moreover, the primer set specific for *Ae. koreicus* confirmed the previous genetic results and showed the presence, at position 203 of our alignment, of the SNP distinct for this species. Six newly generated DNA sequences were deposited in the GenBank database under the accession numbers MH321447-MH321452.

Eight *Ae. koreicus* female specimens were caught at the same sites (site GE6: *n* = 6; GE7: *n* = 2) as of 28th September and 12th October, respectively. A single female specimen, though visibly damaged but still resembling *Ae. koreicus*, was caught on 1st September at site GE3, located 5.7 km from the closest positive site GE6. Unfortunately, DNA amplification failed in this case and genetic confirmation could not be achieved. The other species collected in the traps positive for *Ae. koreicus* in 2016 were: *Culex pipiens* (*n* = 617; 84.2%), *Ae. albopictus* (*n* = 49; 6.7%), *Culiseta longiareolata* (*n* = 9; 1.2%), *Culex* sp. (*n* = 46; 6.3%), and *Aedes* sp. (*n* = 2; 0.3%). The *Ae. koreicus* female specimens collected in 2016 were not analysed for *Flavivirus* detection. Following the first *Ae. koreicus* captures in June–July 2016, one supplementary trapping session using a BG-sentinel trap was performed in August at site GE17, not usually monitored for adult mosquitoes but located in the same quarter of the city; no *Ae. koreicus* specimen was caught. Potential breeding sites were also searched and inspected at sites GE6 and GE7; they consisted of an abandoned plastic container (GE6) and seven manholes (two at GE6, five at GE7). Only *Cx. pipiens* and *Ae. albopictus* larvae were found.

Based on these findings, a retrospective investigation of the 2015 photographic database revealed three damaged *Aedes* sp. samples (one male and two females) similar to *Ae. japonicus*/*Ae. koreicus* in several morphological characters, caught in a BG-sentinel trap located at the Genoa International airport (site GE1) on 3rd and 30th September, respectively. As the male specimen was still preserved, frozen at − 80 °C, a biomolecular assay for the *nad*4 gene performed as previously described obtained an identity of 99% with sequences of *Ae. koreicus*, as was determined for the samples collected in 2016.

Site GE1 is located 2.47 km from the closest positive site in 2016 (GE7) (Fig. 1). Other mosquito specimens collected in the BG-sentinel trap, together with the *Ae. koreicus* male individual, included 91 *Cx. pipiens* and 25 *Ae. albopictus*. The two damaged *Aedes* sp. female specimens were negative at biomolecular assays for *Flavivirus* detection.

In 2017, a total of 3436 adult mosquitoes, including 1073 *Aedes* spp., were collected in Genoa. On 4th May 2017, an adult *Ae. koreicus* male specimen was caught in a gravid trap at site GE6, while an *Aedes* sp. damaged male was trapped in a gravid trap in June at site GE18 (not monitored for adult mosquitoes the previous years). Because the latter site, located at the Flower Market, lies 8.5 km inland from the closest site that tested positive in 2016–2017 (site GE6), genetic analysis was performed to exclude misidentification with the morphologically similar *Ae. japonicus*. The male individual from GE18 was confirmed as *Ae. koreicus* by the mtDNA *nad*4 assay. Furthermore, 33 *Ae. koreicus* individuals (7 males and 26 females) were trapped at eight sites (GE2, GE3, GE5, GE6, GE7, GE15, GE17 and GE18) between July and October 2017. The other mosquito species collected in the *Ae. koreicus*-positive traps in 2017 were: *Cx. pipiens* (*n* = 556; 52.7%), *Cx. hortensis* (*n* = 2; 0.2%), *Cx. territans* (*n* = 1; 0.1%), *Culex* sp. (*n* = 119; 11.3%), *Ae. albopictus* (*n* = 321; 30.4%), *Cs. longiareolata* (*n* = 15; 1.4%), *Cs. annulata* (*n* = 1; 0.1%), *Aedes* (*Ochlerotatus*) sp. (*n* = 1; 0.1%), *Anopheles maculipennis* (*n* = 1; 0.1%), and *Anopheles* sp. (*n* = 3; 0.3%).

Between June and October 2018, 5417 mosquitoes, including 41 *Ae. koreicus* specimens, were collected in Genoa. All four sites found positive for *Ae. koreicus* (GE3, GE5, GE6 and GE18) were monitored by gravid traps and had already been found positive in previous year (GE6 the two previous years). Other species collected in *Ae. koreicus*-positive traps in 2018 were: *Cx. pipiens* (*n* = 568; 63.7%), *Cx. hortensis* (*n* = 1; 0.1%), *Culex* sp. (*n* = 25; 2.8%), *Ae. albopictus* (*n* = 242; 27.2%), *Cs. longiareolata* (*n* = 11; 1.2%), *Cs. annulata* (*n* = 1; 0.1%), *Anopheles plumbeus* (*n* = 1; 0.1%) and *Anopheles* sp. (*n* = 1; 0.1%).

Overall, 87 *Ae. koreicus* adult specimens were collected in Genoa in the four-year period 2015–2018 (Table 3), accounting for 0.5% of all mosquitoes and 1.04% of *Aedes* sp. mosquitoes (Table 4).

**Table 3:** Total number of *Aedes koreicus* adult specimens trapped in Genoa by month (September 2015 to June 2018)

Year	Month	Site ID	Site	Trap type	Females	Males	Sex not determined	Total	Genetically confirmed
2015	9	GE1	Airport	BG-sentinel	0	1	0	1	Y
2016	6	GE7	City centre	BG-sentinel	1	0	0	1	Y
2016	6	GE6	Hospital	Gravid	1	0	0	1	Y
2016	7	GE6	Hospital	Gravid	1	0	0	1	N
2016	8	GE6	Hospital	Gravid	1	0	0	1	Y
2016	8	GE7	City centre	BG-sentinel	0	0	1	1	Y
2016	9	GE6	Hospital	Gravid	3	0	0	3	N
2016	9	GE6	Hospital	Gravid	1	0	0	1	Y
2016	10	GE7	City centre	BG-sentinel	1	0	0	1	N
2017	5	GE6	Hospital	Gravid	0	1	0	1	N
2017	6	GE18	Flower Market	Gravid	0	1	0	1	Y
2017	7	GE3	Hospital	Gravid	0	1	0	1	Y
2017	7	GE6	Hospital	Gravid	4	1	0	5	N
2017	8	GE6	Hospital	Gravid	2	1	0	3	N
2017	8	GE18	Flower Market	Gravid	3	1	0	4	N
2017	8	GE6	Hospital	Gravid	1	0	0	1	N
2017	8	GE15	School	Gravid	2	0	0	2	N
2017	9	GE6	Hospital	Gravid	1	0	0	1	N
2017	9	GE6	Hospital	Gravid	1	1	0	2	N
2017	9	GE2	Port	BG-sentinel	1	0	0	1	N
2017	10	GE3	Hospital	Gravid	3	0	0	3	N
2017	10	GE6	Hospital	Gravid	1	0	0	1	N
2017	10	GE18	Flower Market	Gravid	3	0	0	3	N
2017	10	GE7	City centre	BG-sentinel	1	0	0	1	N
2017	10	GE17	Cemetery	BG-sentinel	1	0	0	1	N
2017	10	GE3	Hospital	Gravid	1	1	0	2	N
2017	10	GE5	Hospital	Gravid	1	0	0	1	N
2017	10	GE6	Hospital	Gravid	0	1	0	1	N
2018	6	GE6	Hospital	Gravid	1	2	0	3	N
2018	6	GE18	Flower Market	Gravid	1	0	0	1	N
2018	6	GE6	Hospital	Gravid	6	2	0	8	N
2018	6	GE18	Flower Market	Gravid	2	0	0	2	N
2018	7	GE18	Flower Market	Gravid	1	0	0	1	N
2018	8	GE6	Hospital	Gravid	9	3	0	12	N
2018	9	GE3	Hospital	Gravid	0	1	0	1	N
2018	9	GE5	Hospital	Gravid	1	0	0	1	N
2018	9	GE6	Hospital	Gravid	4	6	0	10	N
2018	10	GE3	Hospital	Gravid	1	0	0	1	N
2018	10	GE6	Hospital	Gravid	1	0	0	1	N
					62	24	1	87	

*Abbreviations*: Y, yes; N, no

**Table 4:** Yearly percentages of *Aedes koreicus* mosquitoes trapped in Genoa (2015–2018)

Year	No. of mosquitoes	No. of. *Aedes* sp.	No. of *Ae. koreicus*	Overall % *Ae. koreicus*	% *Ae. koreicus vs Aedes* sp.
2015	4147	1802	1	0.02	0.06
2016	4499	1862	10	0.22	0.54
2017	3436	1073	35	1.02	3.26
2018	5417	3646	41	0.76	1.12
Total	17,499	8383	87	0.50	1.04

**Fig. 2:**
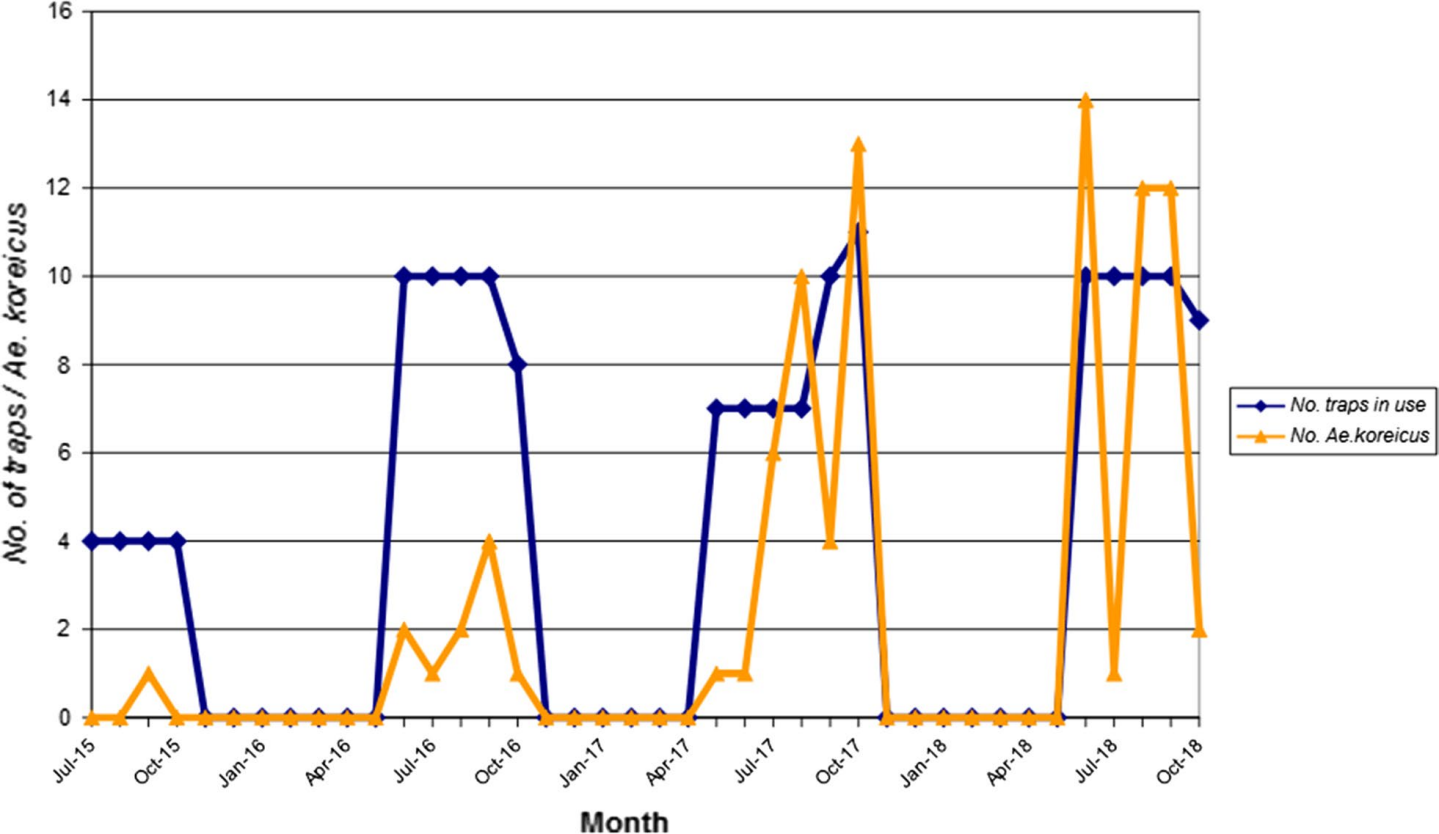
Number of *Aedes koreicus* collected per month *vs* number of traps set for adult mosquitoes

The highest percentages were recorded for 2017, with *Ae. koreicus* specimens accounting for 1.02 and 3.26% of all mosquitoes and of *Aedes* sp., respectively. Table 1 and Fig. 2 show comparative data for the number of *Ae. koreicus* trapped per month *vs* the sampling set up, i.e. the number of specimens caught per trap night and the number of traps in use each month. The highest capture rate was recorded at site GE6. Trapping data indicate a clear increase in the number of *Ae. koreicus* specimens caught in 2016–2017 (Fig. 2) and an expansion of the colonized urban area, with sites GE2, GE3 and GE5 found positive for the species in 2017, albeit negative the previous year (Table 1). In 2018, sites GE2 and GE7 became negative again, despite the increased number of trap nights, which might indicate local small-scale extinctions. The data for 2018, however, confirm the presence of the species in a few key areas, particularly sites GE6 (Villa Scassi Hospital) and GE18 (Flower Market). Gravid and BG-sentinel traps provided 0.83 *vs* 0.27 adults per trap night at positive sites, respectively.

All female specimens analysed for *Flavivirus* detection (*n* = 26 in 2017, grouped in 15 pools; *n* = 27 in 2018, grouped in 11 pools) tested negative.

### *Aedes* spp. eggs

A total of 885, 6397, 12,388 and 10,308 *Aedes* sp. eggs were collected in ovitraps in Genoa in 2015, 2016, 2017 and 2018, respectively, and reared at the IZSPLV laboratory. Ovitrap data are reported in Table 5. In 2015, 2016 and 2018, only *Ae. albopictus* specimens were obtained. Some eggs collected at site GE18 in July and August 2017 appeared unusually shaped on visual inspection, i.e. slightly longer than normal *Ae. albopictus* eggs; 62 of them hatched and the specimens were identified as *Ae. koreicus* at the fourth-instar larval stage (*n* = 8) or at the adult stage (*n* = 54; 29 males and 25 females). One more adult specimen (one female) was obtained from eggs collected at site GE3 on 27 July.

**Table 5:** Sites monitored by ovitraps and *Aedes* sp. eggs collected in Genoa (July 2015 to October 2018)

Site ID	Site	Coordinates	2015 (July–October)			2016 (June–October)			2017 (May–October)			2018 (June–October)		
			Trap type	No. of trap CS	Mean no. of eggs/CS	Trap type	No. of trap CS	Mean no. of eggs/CS	Trap type	No. of trap CS	Mean no. of eggs/CS	Trap type	No. of trap CS	Mean no. of eggs/CS
GE1	Airport	44.4175N, 8.8544E	OT	7	58.14	OT	10	61.10	OT	7	54.29	OT	9	140.78
GE2	Port	44.4069N, 8.9084E	OT	7	68.71	OT	10	37.60	OT	2	41.50	OT	9	70.11
GE3	Hospital	44.4107N, 8.9711E	OT	2	14.50	OT	10	27.00	OT	9	39.33	OT	5	115.80
GE4	Hospital	44.3916N, 8.9896E	OT	2	227.00	OT	10	40,60	OT	8	49.75	OT	5	77.40
GE5	Hospital	44.4008N, 8.9414E	–	–	–	OT	10	64.50	OT	10	185.40	OT	5	465.40
GE6	Hospital	44.4160N, 8.8976E	–	–	–	OT	10	83.90	OT	6	102.67	OT	6	220.67
GE7	City centre	44.4123N, 8.8847E	–	–	–	OT	8	82.25	–	–	–	OT	9	19.11
GE8	City centre	44.4094N, 8.8905E	–	–	–	–	–	–	–	–	–	–	–	–
GE9	City centre	44.4168N, 8.9556E	–	–	–	–	–	–	–	–	–	–	–	–
GE10	Cemetery	44.4298N, 8.9497E	–	–	–	–	–	–	–	–	–	OT	9	41.22
**GE14**	**Urbanized hills**	**44.4170N, 8.8736E**	–	–	–	**OT**	**9**	**53.00**	OT	**8**	**55.88**	–	–	–
GE15	City centre	44.4160N, 8.8856E	–	–	–	OT	8	83.50	OT	7	165.14	–	–	–
**GE16**	**City centre**	**44.4121N, 8.8968E**	–	–	–	**OT**	**8**	**41.88**	**OT**	**8**	**84.50**	–	–	–
GE17	Cemetery	44.4210N, 8.9023E	–	–	–	OT	9	101.11	OT	10	207.40	–	–	–
GE18	Flower Market	44.4898N, 8.8984E	–	–	–	OT	6	30.00	OT	11	202.64	OT	1	499.00
**GE19**	**River area**	**44.4605N, 8.9023E**	–	–	–	**OT**	**4**	**0.00**	**OT**	**4**	**22.75**	–	–	–
**GE20**	**River area**	**44.4255N, 8.8857E**	–	–	–	–	–	–	**OT**	**5**	**39.40**	**OT**	**2**	**182.50**
**GE21**	**Urbanized hills**	**44.4034N, 8.9730E**	–	–	–	–	–	–	**OT**	**6**	**95.83**	**OT**	**2**	**601.00**
GE22	Port	44.4280N, 8.7726E	–	–	–	–	–	–	–	–	–	OT	9	187.11

*Note*: Sites monitored by ovitraps only are highlighted in bold

*Abbreviations*: OT, ovitrap; CS, collection sessions

### Climatic data

**Fig. 3:**
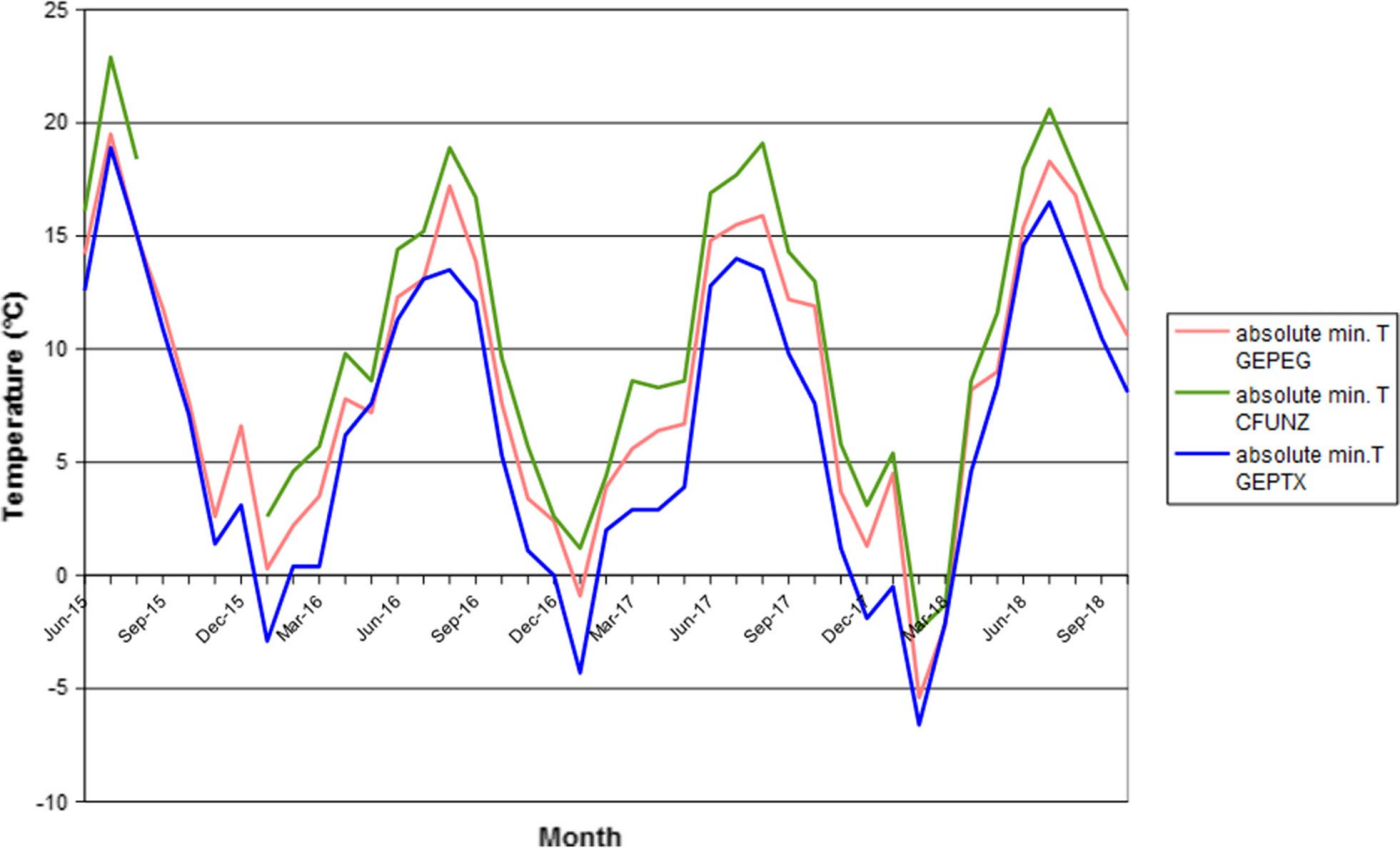
Monthly absolute minimum temperatures recorded at the three automated weather stations

**Fig. 4:**
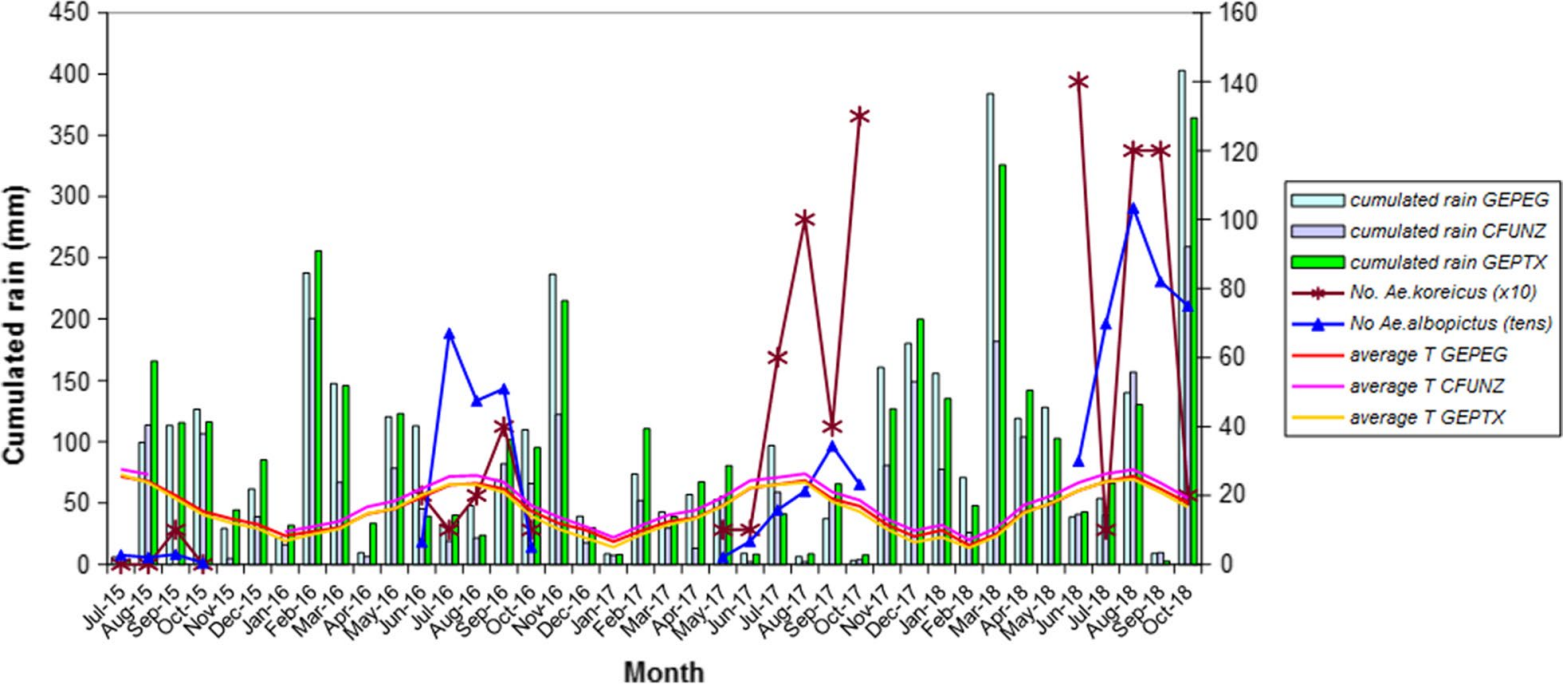
Trapping data for *Ae. koreicus* and *Ae. albopictus vs* climatic data (2015–2018)

Monthly absolute minimum temperature values recorded for the four-year period 2015–2018 at the three weather stations are presented in Fig. 3. Station GEPTX, located in front of the Flower Market, recorded the lowest temperatures during the whole period. Winter 2017–2018 resulted to be the coldest, with − 5.4 °C and − 6.6 °C values recorded in February at stations GEPEG and GEPTX, respectively. In June 2018, *Ae. koreicus* was still present at site GE18, located in front of the GEPTX weather station, where the lowest winter temperatures had been recorded. Cumulated rainfall and average temperature *vs* mosquito trapping data (*Ae. koreicus* and *Ae. albopictus*) are reported in Fig. 4. A clear pattern of association between precipitation trend and trapping success is evident for 2017 and partially for 2018, when adult *Ae. koreicus* were caught during the months with less precipitation preceded by months with higher precipitation.

## Discussion

To our knowledge, the present findings represent the first report of the IMS *Aedes koreicus* in northwest Italy, four years after the species was first detected in northeast Italy. *Aedes koreicus* seems to have successfully survived three consecutive winters in Genoa. Although one or more re-invasions after winter extinctions cannot be excluded, they seem unlikely since the species has been found at the same sites over several consecutive years, e.g. three years at site GE6.

Experimental studies on other IMS of the genus *Aedes*, for instance *Ae. albopictus* and *Ae. aegypti*, stressed the ecological importance of absolute minimum temperatures in limiting expansion of the species [32, 33]. In general, monthly mean and absolute minimum air temperatures in the invaded areas in northeast Italy are similar to those recorded for the native area of *Ae. koreicus* in Korea [10]. Temperatures recorded for Genoa support species survival in the area through the winters of 2015–2016, 2016–2017 and 2017–2018.

In our study, both trap types, gravid and BG-sentinel, were found effective in trapping *Ae. koreicus* specimens of both sexes. Apparently, the former type was more effective than the BG-sentinel trap, as reported by a previous study in northeast Italy [34]. As no trap comparative experiments between trap types were performed, this consideration is purely speculative. In Belgium, gravid traps appeared to be more effective than BG-sentinel traps [9]. Conversely, ovitraps were not the best tool for the early detection of *Ae. koreicus* arrival in Liguria, as similarly observed for the detection of *Ae. japonicus* in the USA [35]. Use of ovitraps allowed detection of the species in Genoa only in the summer of 2017 and at sites that were already positive for adult specimens the previous year. If based on ovitraps alone, mosquito surveillance in the area would have incurred a two-year delay in the discovery of the species.

By establishing an archive for retrospective studies, we were able to date back the presence of *Ae. koreicus* to September 2015. Archives for future investigations can be easily established by preserving male specimens, which are not used for monitoring the circulation of mosquito-borne diseases, as well as female specimens that did not undergo biomolecular assay. Performing genetic analysis to confirm species identity in case of doubtful and/or damaged specimens can also be recommended, especially at sites at high risk of the introduction of IMS.

Regarding the dispersal of *Ae. koreicus*, the average shift of the invaded area centroid in northeast Italy between 2011 and 2014 proceeds at 8 km/year [36]. In Genoa, this is apparently consistent with a dispersal pathway and timing from the city centre (Sampierdarena quarter) to the north, along the Polcevera valley, to the Flower Market area (Pontedecimo quarter, i.e. site GE18), or *vice versa* (a distance of *c.*8.5 km). Pontedecimo and the city centre are connected by railway, roadway and highway. As road traffic can play a role in the long-range dispersal of IMS [6], introduction of the species into Liguria from north or northeast Italy, where the presence of *Ae. koreicus* has been documented since 2011 and its range is still expanding, cannot be ruled out. However, given the presence of an international airport and a very busy commercial port, any of these other areas may represent the first site of introduction. Interestingly, however, no other *Ae. koreicus* specimens were found at the airport site (GE1) after September 2015.

A study on *Ae. albopictus* calculated an average total dispersal distance of 3.6–4.6 km/year/generation in Italy, with a passive dispersal component of 2.8–4.1 km/year/generation [37]. The number of generations per year that can be completed by *Ae. koreicus* in Europe remains unknown. However, a laboratory-based study under different temperature conditions performed on specimens of the ecologically similar *Ae. japonicus japonicus* collected in Germany calculated an average of 4.72 potential generations per year [38]. Should this be applicable to *Ae. koreicus*, we estimate a theoretical average total dispersal distance of 17.0–21.7 km/year in favourable conditions.

Figure 4 presents the trend of trapping data for *Ae. koreicus* and *Ae. albopictus vs* climatic data. To allow visual comparison of the data trends, trapping data are not given to scale: *Ae. koreicus* data are shown 10-fold higher and *Ae. albopictus* 10-fold lower than the actual values.

*Aedes koreicus* trapping data in 2016–2018 followed a bimodal trend, unlike the congeneric *Ae. albopictus*, although there was no difference in the trapping set up for the two species (Figs. 2, 4). The trend for *Ae. koreicus* might be explained by less trapping operated at positive sites in September 2017 (one trap night only at site GE7) and in July 2018 (one trap night only at site GE6) (Table 5). However, despite the lower effort in August 2018 (one trap night only at site GE6), the number of *Ae. koreicus* specimens trapped was comparably high.

Concerning interspecific interactions, larval competition between *Ae. albopictus* and *Ae. koreicus* under natural conditions needs to be investigated. In a laboratory-based study, weak larval competition was observed, with a slight advantage of the former owing to their faster development [39]. *Aedes koreicus* seems to take advantage of higher altitudes and lower temperatures, as noted for northeast Italy, where its presence has been recorded at elevations up to 1250 masl [11]. All sites in Genoa that were positive for *Ae. koreicus* were also positive for *Ae. albopictus*. However, as all the sites monitored during this study were located at less than 100 masl, colonization at higher elevations by *Ae. koreicus* in the absence of *Ae. albopictus* cannot be ruled out.

Finally, no flaviviral infection in *Ae. koreicus* was detected during our study; this might be explained by the low density of the species in the sampling area and the consequent low encounter rate with infected hosts.

## Conclusions

Entomological surveillance of sites at risk in northwest Italy allowed for the early detection of the IMS *Ae. koreicus* in Liguria. This is, to our knowledge, the first report of the species in the area, and in a port city in the Mediterranean basin. So far, this invasive mosquito species appears to be present at low densities in most of the urban area of Genoa. Both morphological features and genetic data support a common origin for the Ligurian and most of the other European populations; however, the ways of entry of this species in Europe remain unknown. Future in-depth genetic analysis, including short tandem repeat (STR) genotyping, would help to determine the exact origin of the European populations and their dispersal pathways.

In Genoa, the species was first detected in September 2015 in the area of the international airport, which is located near the commercial port. Either the former or the latter might be the site of introduction, but this cannot be demonstrated. The finding of *Ae. koreicus* adults and eggs at the Flower Market in 2017, which lies inland more than 8 km from the closest positive site in 2016 and 2017, but was not monitored for adult specimens in the previous years, raises questions about how the species was introduced in Liguria and how much of the area is already invaded, which might be much greater than observed so far. To date, however, no other monitored sites throughout Liguria have been found positive for the species. Although multiple introduction events cannot be excluded, *Ae. koreicus* seems to have overwintered at least three times in the city and has become established in key areas. There were clear indications of species expansion in the city in 2017. Regarding monitoring tools, ovitraps were less effective for monitoring the species than the traps for adult mosquitoes. Although all *Ae. koreicus* pools analyzed were negative for flaviviruses, virological surveillance on this IMS still makes sense, given its potential to act as a vector of mosquito-borne diseases. Mosquito surveillance in Liguria, with a particular focus on Genoa city, will be continued in 2019. Given the importance of Genoa as a commercial and tourism hub, further and accelerated spread of the species in Italy and beyond can be expected, as was observed for the Asian tiger mosquito *Ae. albopictus*. Unfortunately, no control measures have been deployed or are foreseen to eradicate the species or slow down its expansion.

## Data Availability

Data supporting the conclusions of this article are included within the article. Eight adult *Aedes koreicus* voucher specimens (4 males and 4 females) obtained from egg rearing during this study are preserved at the Museo Civico di Storia Naturale in Genoa, Italy (MSNG) as reference material. *nad*4 gene sequences obtained from six specimens were deposited in GenBank under the accession numbers MH321447–MH321452.

## Abbreviations

IMS: invasive mosquito species
masl: meters above sea level

## References

[1] Schaffner F, Medlock JM, Van Bortel W (2013). Public health significance of invasive mosquitoes in Europe. Clin Microbiol Infect.

[2] Adhami J, Reiter P (1998). Introduction and establishment of *Aedes* (*Stegomyia*) *albopictus* Skuse (Diptera: Culicidae) in Albania. J Am Mosq Control Assoc.

[3] Sabatini A, Raineri V, Trovato G, Coluzzi M (1990). *Aedes albopictus* in Italy and possible diffusion of the species into the Mediterranean area. Parassitologia.

[4] Angelini R, Finarelli AC, Angelini P, Po C, Petropulacos K, Silvi G (2007). Chikungunya in northeastern Italy: a summing up of the outbreak. Euro Surveill.

[5] Venturi G, Di Luca M, Fortuna C, Remoli ME, Riccardo F, Severini F (2017). Detection of a chikungunya outbreak in Central Italy, August to September 2017. Euro Surveill.

[6] Medlock JM, Hansford KM, Schaffner F, Versteirt V, Hendrickx G, Zeller H, Van Bortel W (2012). A review of the invasive mosquitoes in Europe: Ecology, public health risk, and control options. Vector Borne Zoonotic Dis.

[7] Capelli G, Drago A, Martini S, Montarsi F, Soppelsa M, Delai N (2011). First report in Italy of the exotic mosquito species *Aedes* (*Finlaya*) *koreicus*, a potential vector of arboviruses and filariae. Parasit Vectors.

[8] Seidel B, Montarsi F, Huemer HP, Indra A, Capelli G, Allerberger F, Nowotny N (2016). First record of the Asian bush mosquito, *Aedes japonicus japonicus*, in Italy: invasion from an established Austrian population. Parasit Vectors.

[9] Versteirt V, de Clercq EM, Fonseca DM, Pecor J, Schaffner F, Coosemans M, Van Bortel M (2012). Bionomics of the established exotic mosquito species *Aedes koreicus* in Belgium, Europe. J Med Entomol.

[10] Montarsi F, Martini S, Dal Pont M, Delai N, Ferro Milone N, Mazzucato M (2013). Distribution and habitat characterization of the recently introduced invasive mosquito *Aedes koreicus* [*Hulecoeteomyia koreica*], a new potential vector and pest in north-eastern Italy. Parasit Vectors.

[11] Montarsi F, Drago A, Martini S, Calzolari M, De Filippo F, Bianchi A (2015). Current distribution of the invasive mosquito species, *Aedes koreicus* [*Hulecoeteomyia koreica*] in northern Italy. Parasit Vectors.

[12] Ciocchetta S, Prow NA, Darbro JM, Frentiu FD, Savino S, Montarsi F (2018). The new European invader *Aedes* (*Finlaya*) *koreicus*: a potential vector of chikungunya virus. Pathog Glob Health.

[13] Werner D, Zielke DE, Kampen H (2015). First record of *Aedes koreicus* (Diptera: Culicidae) in Germany. Parasitol Res.

[14] Suter T, Flacio E, Feijoò Fariña B, Engeler L, Tonolla M, Muller P (2015). First report of the invasive mosquito species *Aedes koreicus* in the Swiss-Italian border region. Parasit Vectors.

[15] Kurucz K, Kiss V, Zana B, Schmieder V, Kepner A, Jakab F, Kemenesi G (2016). Emergence of *Aedes koreicus* (Diptera: Culicidae) in an urban area, Hungary. Parasitol Res.

[16] Kalan K, Susnjar J, Ivovic V, Buzan E (2017). First record of *Aedes koreicus* (Diptera, Culicidae) in Slovenia. Parasitol Res.

[17] Pfitzner WP, Lehner A, Hoffmann D, Czajka C, Becker N (2018). First record and morphological characterization of an established population of *Aedes* (*Hulecoeteomyia*) *koreicus* (Diptera: Culicidae) in Germany. Parasit Vectors.

[18] Medlock JM, Hansford KM, Versteirt V, Kull B, Kampen H, Fontenille D (2015). An entomological review of invasive mosquitoes in Europe. Bull Entomol Res.

[19] Schaffner F, Bellini R, Petric D, Scholte EJ (2009). The invasive mosquito *Aedes japonicus* in central Europe. Med Vet Entomol.

[20] Miles JA (1964). Some ecological aspects of the problem of arthropod-borne animal viruses in the Western Pacific and South-East Asia regions. Bull World Health Organ.

[21] Kurucz K, Kiss V, Zana B, Jacab F, Kemenesi G (2018). Filarial nematode (order: Spirurida) surveillance in urban habitats, in the city of Pécs (Hungary). Parasitol Res.

[22] Feng LC (1938). The tree hole species of mosquitoes of Peiping, China. Chin Med J.

[23] Montarsi F, Ciocchetta S, Devine G, Ravagnan S, Mutinelli F, Frangipane di Regalbono A (2015). Development of *Dirofilaria immitis* within the mosquito *Aedes* (*Finlaya*) *koreicus*, a new invasive species for Europe. Parasit Vectors.

[24] Sambri V, Capobianchi M, Charrel R, Fyodorova M, Gaibani P, Gould E (2013). West Nile virus in Europe: emergence, epidemiology, diagnosis, treatment, and prevention. Clin Microbiol Infect.

[25] Fortuna C, Remoli ME, Rizzo C, Benedetti E, Fiorentini C, Bella A (2017). Imported arboviral infections in Italy, July 2014–October 2015: a National Reference Laboratory report. BMC Infect Dis.

[26] Severini F, Toma L, Di Luca M, Romi R (2009). Le zanzare italiane: generalità e identificazione degli adulti (Diptera, Culicidae). Fragmenta Entomol.

[27] European Centre for Disease Prevention and Control (ECDC) (2012). Guidelines for the surveillance of invasive mosquitoes in Europe.

[28] Ree H (2003). Taxonomic review and revised keys of the Korean mosquitoes (Diptera: Culicidae). Korean J Entomol.

[29] Fonseca DM, Campbell S, Crans WJ, Mogi M, Miyagi I, Toma T (2001). *Aedes* (*Finlaya*) *japonicus* (Diptera: Culicidae), a newly recognized mosquito in the United States: analyses of genetic variation in the United States and putative source populations. J Med Entomol.

[30] Cameron EC, Wilkerson RC, Mogi M, Miyagi I, Toma T, Kim HC (2010). Molecular phylogenetics of *Aedes japonicus*, a disease vector that recently invaded western Europe, North America, and the Hawaiian islands. J Med Entomol.

[31] Scaramozzino N, Crance J-M, Jouan A, DeBriel DA, Stoll F, Garin D (2001). Comparison of flavivirus universal primer pairs and development of a rapid, highly sensitive heminested reverse transcription-PCR assay for detection of flaviviruses targeted to a conserved region of the NS5 gene sequences. J Clin Microbiol.

[32] Hanson SM, Craig GB (1995). Relationship between cold hardiness and supercooling point in *Aedes albopictus* eggs. J Am Mosq Control Assoc.

[33] Thomas SM, Obermayr U, Fischer D, Kreyling J, Beierkuhnlein C (2012). Low-temperature threshold for egg survival of a post-diapause and non-diapause European aedine strain, *Aedes albopictus* (Diptera: Culicidae). Parasit Vectors.

[34] Baldacchino F, Montarsi F, Arnoldi D, Barategui C, Ferro Milone N, Da Rold G (2017). A 2-yr mosquito survey focusing on *Aedes koreicus* (Diptera: Culicidae) in northern Italy and implications for adult trapping. J Med Entomol.

[35] Andreadis TG, Anderson JF, Munstermann LE, Wolf RJ, Florin DA (2001). Discovery, distribution, and abundance of the newly introduced mosquito *Ochlerotatus japonicus* (Diptera: Culicidae) in Connecticut, USA. J Med Entomol.

[36] Marcantonio M, Metz M, Baldacchino F, Arnoldi D, Montarsi F, Capelli G (2016). First assessment of potential distribution and dispersal capacity of the emerging invasive mosquito *Aedes koreicus* in northeast Italy. Parasit Vectors.

[37] Trájer A, Hammer T, Kacsala I, Tánczos B, Bagi N, Padisák J (2017). Decoupling of active and passive reasons for the invasion dynamics of *Aedes albopictus* Skuse (Diptera: Culicidae): comparisons of dispersal history in the Apennine and Florida peninsulas. J Vector Ecol.

[38] Reuss F, Wieser A, Niamir A, Bálint M, Kuch U, Pfenninger M, Müller R (2018). Thermal experiments with the Asian bush mosquito (*Aedes japonicus japonicus*) and implications for its distribution in Germany. Parasit Vectors.

[39] Baldacchino F, Montarsi F, Arnoldi D, Barategui C, Ferro Milone N, Da Rold G (2017). Weak larval competition between two invasive mosquitoes *Aedes koreicus* and *Aedes albopictus* (Diptera: Culicidae). J Med Entomol.

